# Impact of Colonoscopy Bowel Preparation on Intestinal Microbiota

**DOI:** 10.1371/journal.pone.0062815

**Published:** 2013-05-01

**Authors:** Claire L. O’Brien, Gwen E. Allison, Florian Grimpen, Paul Pavli

**Affiliations:** 1 Australian National University Medical School, Canberra, Australia; 2 Department of Gastroenterology and Hepatology, Royal Brisbane and Women’s Hospital, Herston, Queensland, Australia; 3 Australian National University Research School of Biology, Canberra, Australia; 4 Gastroenterology and Hepatology Unit, The Canberra Hospital, Canberra, Australia; University of Waterloo, Canada

## Abstract

The gut microbiota is important in maintaining human health, but numerous factors have the potential to alter its composition. Our aim was to examine the impact of a standard bowel preparation on the intestinal microbiota using two different techniques. Fifteen subjects undergoing colonoscopy consumed a bowel preparation comprised of 10 mg bisacodyl and 2 L polyethylene glycol. The microbiota of stool samples, collected one month before, one week before (pre-colonoscopy), and one week, one month, and three to six months after colonoscopy (post-colonoscopy) was evaluated. Two samples were taken three to six months apart from five healthy subjects who did not undergo colonoscopy. Universal primers targeting the V2–V3 region of the 16S rRNA gene were used to PCR amplify all samples for denaturing gradient gel electrophoresis (PCR-DGGE). Pre- and post-colonoscopy samples were compared using Dice’s similarity coefficients. Three samples from ten subjects who underwent colonoscopy, and both samples from the five subjects who didn’t, were used for high-throughput sequencing of the V1–V3 region of the 16S rRNA gene. Samples were curated and analysed in Mothur. Results of the DGGE analyses show that the fecal microbiota of a small number of subjects had short-term changes. High-throughput sequencing results indicated that the variation between the samples of subjects who underwent colonoscopy was no greater than the variation observed between samples from subjects who did not. We conclude that bowel preparation does not have a lasting effect on the composition of the intestinal microbiota for the majority of subjects.

## Introduction

The microbial community of the human gastrointestinal tract is unique to each individual [1], has a stable component [2], [3], contributes to maturation of the host immune system [4], [5] and is important in maintaining human health [6]. The development of non-culture-based molecular approaches to the study of the human intestinal microbiota has led to a greater understanding of its complexity both at an individual and population level [7]. There are numerous factors that have the potential to alter the microbiota’s composition: the use of broad-spectrum antibiotics perturbs the gut microbiota and it may take weeks, months, even years, for it to return to its pre-exposure state [8], [9]. Diet may also influence the intestine’s microbial composition [6], [10], [11].

Colonoscopy is performed commonly in our community. It requires that the bowel be emptied of its contents, a process facilitated by the ingestion of an oral laxative preparation. Two studies have investigated the effects of a colonoscopy preparation on the gut microbiota [12], [13]. Mai *et al*. [12] used PCR-DGGE and 16S rRNA gene *E. coli*-clone libraries to compare fecal samples in five subjects: one specimen pre-colonoscopy was compared to two samples obtained 2–4 and 6–8 weeks post-colonoscopy. For three out of five patients the two post-colonoscopy DGGE results were more similar to each other than the pre-colonoscopy sample and the authors concluded that the composition of the microbiota is disturbed in patients undergoing colonoscopy.

Harrell *et al.* [13] assessed the short-term effects of a standard colonoscopy preparation (polyethylene glycol, PEG) on the mucosa-associated microbiota obtained from biopsies from a total of 12 healthy individuals who underwent two sigmoidoscopies. The first phase of the study produced divergent results (diversity increased in 3/5 and decreased in 2/5 subjects), so they controlled for the effects of time and a 24-hour clear liquid diet in a second phase of 2–3 subjects per group. They concluded that the PEG preparation, not the passage of time nor the liquid diet, altered the colonic microbiota short-term.

The aim of this study was to determine whether or not the composition of the fecal microbiota changes short- or long-term after colonoscopy. We studied 20 subjects using PCR-DGGE to compare the representation of the major species, and high-throughput 16S rRNA gene sequencing to quantitate differences in the total microbiota in a subset of 15 subjects.

## Methods

### Ethics Statement

Written consent was given by each subject and ethical approval was granted by ACT Health (protocol #: ETH.6/03.276) and the Australian National University (protocol #: 2007/131[LESC-CMHS]).

### Subject Characteristics

Twenty subjects (10 males, 10 females), with an average age of 58 y (range 46–69 y), were evaluated. No subject consumed antibiotics in the six months prior the study. Fifteen subjects ingested 10 mg bisacodyl and 2 L polyethylene glycol (PEG) and underwent colonoscopy for a range of indications, five did not. For those who consumed the preparation, fecal samples were collected one month before (−1m), one week before (−1w), and where possible, within one week (+1w), one month (+1m), and three to six months (+3m) after the procedure. An average of 3.7 samples was taken from each subject that underwent the procedure. For those who did not consume the preparation, two samples were collected three to six months apart. These subjects and their first sample are refered to as A, B, C H and W, and their second sample denoted +3m. All samples were frozen immediately after collection, and stored at −80°C until required.

All samples from all subjects were analysed by polymerase chain reaction – denaturing gradient gel electrophoresis (PCR-DGGE); samples from 10 subjects who consumed the preparation, with various PCR-DGGE results, were subsequently analysed by high-throughput sequencing (HTS). For HTS, samples −1m and −1w, and the last available sample for each of these subjects (either sample +1w, +1m, or +3m) were selected. Both samples from the five subjects who did not consume the preparation were also subjected to HTS analysis.

### DNA Extraction and Quantification

As bacteria are not evenly distributed throughout stool samples [14], material was collected from eight different sites from each specimen, combined and used for the DNA extraction. Total DNA was extracted using the QIAamp DNA Stool Mini-kit according to the manufacturer’s instructions (Qiagen). Bacterial cell lysis was conducted at 95°C for 5 min, and proteinase K incubation at 70°C for 20 min. DNA was quantified using a Nanodrop® ND-1000 spectrophotometer (Analytical Technologies).

### PCR-DGGE

The microbiota of the different samples was compared using the universal bacterial PCR-DGGE protocol of Walter *et al.* [15], with a few modifications. DNA was amplified using primers targeting the V2–V3 region of the 16S ribosomal RNA gene of bacteria (positions 339–539 of the *E. coli* 16S rRNA gene), and were as follows: HDA-1-GC (**CGC CCG GGG CGC GCC CCG GGC GGG GCG GGG GCA CGG GGG G**AC TCC TAC GGG AGG CAG CAG T-3′) (GC clamp in bold), and HDA-2 (5′-GTA TTA CCG CGG CTG CTG GCA C-3′). The 50 µl PCR reactions contained 20 ng template DNA, 1.25 U of *Taq* (Amplitaq), 2.0 mM MgCl_2_, 5 µl 10×buffer, 0.2 mM of each deoxynucleoside triphosphate (Promega), sterile water, and 10 pmol of each primer. Amplification conditions were as follows: 1 cycle at 94°C for 4 min; 30 cycles of 94°C, 30 s; 56°C, 30 s; 68°C, 1 min, and 1 cycle at 63°C for 7 min. The intensity of the PCR product on agarose gels was used as a guide to determine the volume loaded on the denaturing gradient gel to ensure equal amounts of PCR product were used. A species ladder, referred to as the marker, comprising 16S rRNA gene amplicons that migrated to various positions, was run in the two end lanes of each gel.

DGGE was performed to separate the HDA-PCR amplicons, using a DCode universal mutation detection system (BioRad). Polyacrylamide gels were prepared and run with 1×TAE buffer (Amresco). The denaturing gradient was created using two 6% acrylamide-bis, 37.5∶1 (BioRad) stock solutions. The polymerisation catalyst was 10% ammonium persulfate (95 µl), the adjunct catalyst TEMED (55 µl) (Amresco). The gels contained a 22 to 55% gradient of urea and formamide increasing in the direction of electrophoresis. The electrophoresis was conducted at a constant voltage of 130 V at 60°C for 4 h. Gels were stained with fresh ethidium bromide solution (5 µg/ml) for 20 min, destained in MilliQ water for 20 min, visualised using an ultraviolet transilluminator and photographed.

For each subject, the PCR products from all fecal samples were run on the same gel to eliminate gel-to-gel differences in DGGE profiles [16], [17]. The gels were loaded into the BioNumerics software package (v 5.10, Applied Maths), normalised against the marker, and the presence/absence of bands compared using a 3% tolerance limit and 2% optimization. Dendrogram and cluster analysis were performed using algorithms within BioNumerics. Percent similarity among different bacterial community profiles belonging to samples within a subject were scored by the Dice coefficient. The Unweighted Pair Group Method with arithmetic means (UPGMA) was used to obtain the dendrograms.

### PCR Amplification and Sequence Analysis for HTS

DNA was amplified using primers targeting the V1–V3 region of the 16S ribosomal RNA gene of bacteria, equivalent to positions 27–518 of the *E. coli* 16S rRNA gene, using the following primers: 27F (5-**CCATCTCATCCCTGCGTGTCTCCGACTCAG** *MID*GAGTTTGATCMTGGCTCAG-3) and 518R (5-**CCTATCCCCTGTGTGCCTTGGCAGTCTCAG**WTTACCGCGGC TGCTGC-3) where the sequences in bold represent the forward primer A and reverse primer B for the GS FLX TitaniumMV em PCR (LibL) v2 kit (Roche), *MID* represents the 8 bp multiplex identifier (MID) and the remainder represents the 16S rRNA gene forward and reverse primers amplifying the V1–V3 regions. The 48 MID sequences were kindly provided by Professor Andrew Benson [18]. The 50 µl PCR reactions contained 20 ng template DNA, 1 U high fidelity Platinum *Taq* DNA polymersase (Invitrogen), 2.0 mM MgSO_4_, 5 µl 10×PCR buffer, 0.2 mM of each deoxynucleoside triphosphate (Promega), sterile water and 10 pmol of each primer. PCR reactions were performed under the following conditions: initial denaturation at 94°C for 3 min, then 30 cycles of: denaturation at 94°C for 15 s, annealing at 55°C for 30 s, and extension at 68°C for 60 s. The final extension was at 68°C for 10 min. The amplicons were extracted from 2% agarose gels, and purified using a Wizard SV Gel & PCR Clean-Up System (Promega) and the Agencourt AMPure XP system (Beckman Coulter). Concentrations were determined using an Agilent 2100 Bioanalyzer (Agilent Technologies) with DNA 1000 chips. Equal amounts of the PCR products were combined (total 500 ng per 48 samples) to complete each library. Emulsion-based PCR amplification and sequencing on the 454 Genome Sequencer FLX-Titanium system was performed at the Biological Research Facility, Canberra, Australia, according to the manufacturer’s instructions (454 Life Sciences, Branford, CT). Signal processing and base calling were performed using the GS FLX+ System’s software (Roche).

Raw sequences of the V1–V3 hypervariable regions of the 16S rRNA gene were curated and processed using the open-source program, Mothur v.1.23.1 [19]; sequences were trimmed of primer and barcode sequences (primer differences allowed, 2 bp, barcodes, 1 bp) and denoised using the shhh.flows command, a translation of Chris Quince’s PyroNoise algorithim [20]. Sequences were aligned using the SILVA database, and chimeras removed using the Uchime code [21]. The taxonomy of sequences was determined using the classify.seqs command with RDP (2010) training sets, 1000 iterations, and a cut-off of 80%. Distance matrices were generated using a cutoff of 0.30. Files containing operational taxonomic units (OTUs) found across all samples (shared files) were used to describe the dissimilarity (1-minus similarity) among all samples. Shared files, in which the proportion of each phyla/OTU in each sample was computed, were used as inputs when calculating distances between all samples. The resulting distance matrices were used when calculating the Jaccard (presence/absence) and Yue and Clayton theta (relative abundance) measures of dissimilarity for the dendrograms and non-metric dimensional scaling (NMDS) plots. The minimum dimensions used for the NMDS calulations was two, the minimum number of iterations was 10, the maximum 500. Two NMDS axes were plotted in Excel. The statistical program JMP (v.9) was used for matched-pairs *t*-tests of the mean distances between samples in the Jaccard and Yue and Clayton theta NMDS plots.

All sff files generated by the 454 Genome Sequencer, their associated metadata, primer and MID sequences, are included in dataset S1.

## Results

### DGGE Analysis

There was an average of 26 16S rRNA gene fragments/bands per DGGE profile. To define normal temporal variation, Dice similarity coefficients were calculated for the DGGE profiles of the two pre-colonoscopy samples from those subjects who underwent colonoscopy, and from both samples from subjects who did not. The average similarity was 93.6% and the range was 86–100% for these 20 subjects (Table 1).

**Table 1:** Dice similarity coefficients (%) of 16S rRNA amplicon DGGE profiles for fecal samples collected pre- and post-colonoscopy bowel preparation.

Sample comparisons					
Subject	−1m *vs* −1w	−1m *vs*+1w	−1m *vs*LS	−1w *vs*+1w	−1w *vs*LS
1	91	94	94*	94*	94*
2	98	**84**	87**	86**	93**
3	91	90	90*	95*	95*
4	94	**83**	**83** *	**83** *	**83** *
5	93	86	91**	**84** **	87**
6	97	93	93*	97*	97*
7	88	94	91**	94**	100**
8	93	93	93*	87*	87*
9	95	† **	**80** **	† **	**80** **
10	86	86	86*	95*	95*
11∧	94	∧	∧	∧	∧
12	88	95	92**	93**	86**
13	95	**84**	**65** **	88**	**71** **
14	98	95	100**	97**	98**
15	100	100	100*	100*	100*
A***	98				
B***	88				
C***	94				
H***	95				
W***	95				
**Average**	**93.6**				

† 1 week post-colonoscopy sample not obtained.

* LS, last sample, obtained 1 week post-colonoscopy.

** LS, last sample, obtained 1 month or 3–6 months post-colonoscopy.

*** Samplesobtained 3–6 months apart, patient did not undergo colonoscopy.

∧ The post-colonoscopy comparisons are invalid for technical reasons.

The similarity between the pre- and last post-colonoscopy samples is also reported in Table 1. For the majority (11/14) of subjects, the similarity co-efficients between a pre-colonoscopy sample and the last available post-colonoscopy sample were within the range of normal temporal variation. Seven subjects had similarity coefficient values outside the estimated range of normal temporal variation for any given sample comparison (Table 1). Those subjects who did not revert back to the pre-colonoscopy state, when sample −1w was compared with the last sample from that patient, had sample comparisons outside the range of normal temporal variation for at least one comparison involving sample +1w (subjects 4, 9, and 13). These three subjects underwent colonoscopy for different indications and were on different or no medications. We therefore sought to determine whether or not there were any unique clinical characteristics that could account for the changes seen in these three subjects before and after colonoscopy (4, 9, 13). Subject 4 was unique relative to the other subjects in that the indication for colonoscopy was low iron, and he had been on iron supplements until 10 days prior to the procedure, which may account for the changes post-procedure. Subject 9 suffers from ulcerative colitis and was on a number of medications relative to two other subjects with UC (3 and 5) who were only on 5ASA and showed no changes pre- and post-colonoscopy. Subject 13 underwent a surveillance colonoscopy for a family history of colorectal cancer and was on no medications. We conclude that the colonoscopy preparation did not significantly change the gut microbiota for the majority of subjects as determined by PCR-DGGE.

### High-throughput Sequencing (HTS) Analysis of Pre- and Post-colonoscopy Samples

A total of 565,174 raw sequences of the V1–V3 hypervariable regions of the 16S ribosomal RNA gene were processed using Mothur v.1.23.1 [19], yielding a total of 339,428 sequences (average of 251 base pairs). Each sample was covered by an average of 8,054 high quality reads (range = 554–16,395) (Table 2). The total number of unique sequences, determined after filtering the alignment, was 19,169. Discordance between observed and estimated richness, determined by Chao (range of 53–73%) and Ace (40–69%), is likely due to the presence of rare species, which occur naturally in large numbers in microbial communities [22], [23]. A summary of observed and estimated richness, diversity, and sample coverage is provided in Table 2.

**Table 2:** Summary of high-throughput sequencing parameters.

Subject	Sample∧	n seqs#	Observed OTUs*	Chao†	Ace†	SHN**	GC$
2	−1m	16395	1032	1673 (1538, 1845)	2389 (2233, 2564)	4.2	0.97
	−1w	9028	701	1145 (1030, 1299)	1473 (1361, 1603)	4.63	0.96
	+3m	13442	1154	1848 (1707, 2027)	2474 (2325, 2642)	5	0.96
3	−1m	6548	354	548 (482, 649)	572 (509, 662)	3.11	0.98
	−1w	8729	371	534 (476, 626)	546 (493, 620)	3.94	0.98
	+1w	7988	435	620 (561, 706)	818 (744, 909)	3.35	0.98
4	−1m	5887	416	679 (596, 800)	928 (838, 1036)	3.47	0.97
	−1w	8525	354	564 (493, 672)	811 (728, 913)	2.3	0.98
	+1w	6700	249	414 (348, 525)	480 (423, 554)	2.07	0.98
5	−1m	8794	661	1082 (972, 1231)	1407 (1294, 1539)	4.2	0.96
	−1w	9071	387	576 (513, 670)	718 (651, 802)	2.53	0.98
	+3m	8830	761	1093 (1009, 1205)	1169 (1083, 1277)	4.7	0.96
7	−1m	9914	565	1068 (925, 1267)	1427 (1304, 1571)	3.85	0.97
	−1w	8842	359	553 (488, 651)	730 (656, 821)	2.55	0.98
	+3m	12470	464	712 (635, 825)	885 (809, 978)	3.44	0.98
8	−1m	901	163	273 (221, 370)	310 (268, 367)	4.28	0.92
	−1w	9240	683	1145 (1029, 1302)	1542 (1421, 1683)	3.81	0.96
	+1w	6505	374	619 (537, 742)	829 (744, 933)	3.59	0.97
9	−1m	8540	442	723 (632, 856)	870 (791, 967)	3.88	0.98
	−1w	8666	567	879 (790, 1002)	914 (832, 1021)	4.14	0.97
	+1m	5882	148	231 (192, 304)	294 (251, 353)	1.55	0.99
11	−1m	9081	523	958 (828, 1145)	1216 (1109, 1341)	4.17	0.97
	−1w	4817	281	404 (354, 486)	404 (363, 466)	4.02	0.98
	+1w	10114	546	853 (766, 972)	889 (807, 996)	3.6	0.97
13	−1m	11892	774	1296 (1169, 1464)	1770 (1638, 1921)	3.92	0.97
	−1w	11634	912	1453 (1331, 1610)	1946 (1812, 2098)	4.65	0.96
	+1m	9829	630	1063 (947, 1220)	1531 (1403, 1679)	3.96	0.97
15	−1m	10265	994	1519 (1403, 1666)	1953 (1829, 2095)	4.98	0.96
	−1w	12091	764	1258 (1136, 1419)	1550 (1437, 1681)	4.02	0.97
	+1w	8683	719	1128 (1025, 1265)	1431 (1325, 1556)	4.35	0.96
A	A	2256	141	239 (192, 327)	346 (291, 420)	2.79	0.97
	A +3m	554	74	120 (95, 176)	138 (105, 205)	2.47	0.93
B	B	8077	226	309 (274, 370)	390 (346, 449)	2.51	0.99
	B +3m	7659	307	459 (404, 545)	475 (423, 548)	1.91	0.98
C	C	1801	239	382 (327, 472)	510 (447, 592)	3.91	0.94
	C +3m	10951	531	827 (737, 957)	976 (897, 1071)	4.02	0.98
H	H	4289	368	611 (530, 731)	797 (717, 897)	3.86	0.96
	H +3m	6551	639	1037 (932, 1180)	1328 (1224, 1450)	4.31	0.95
W	W	6617	344	542 (472, 649)	536 (480, 616)	3.31	0.98
	W +3m	4116	499	852 (750, 996)	1052 (957, 1167)	4.31	0.94
Average:	–	8054	504	–	–	3.64	0.96

# number of quality sequences obtained for a given sample.

* number of observed operational taxonomic units.

** Shannon diversity index.

$ Good’s coverage; C = 1 n_1_/N, where n_1_ is the number of OTUs that have been sampled once, and N is the total number of sequences.

† Calculated with Mothur at the 3% distance level. Values in brackets are 95% confidence intervals as calculated by Mothur.

∧ For subjects who underwent colonoscopy: −1m, −1w, sample obtained one month, one week pre-colonoscopy, +1w, +1m, +3m, sample obtained 1 week, one month, or 3–6 months post-colonoscopy, respectively. For subjects who did not undergo colonoscopy: +3m, sample obtained 3–6 months after the first sample.

**Figure 1:**
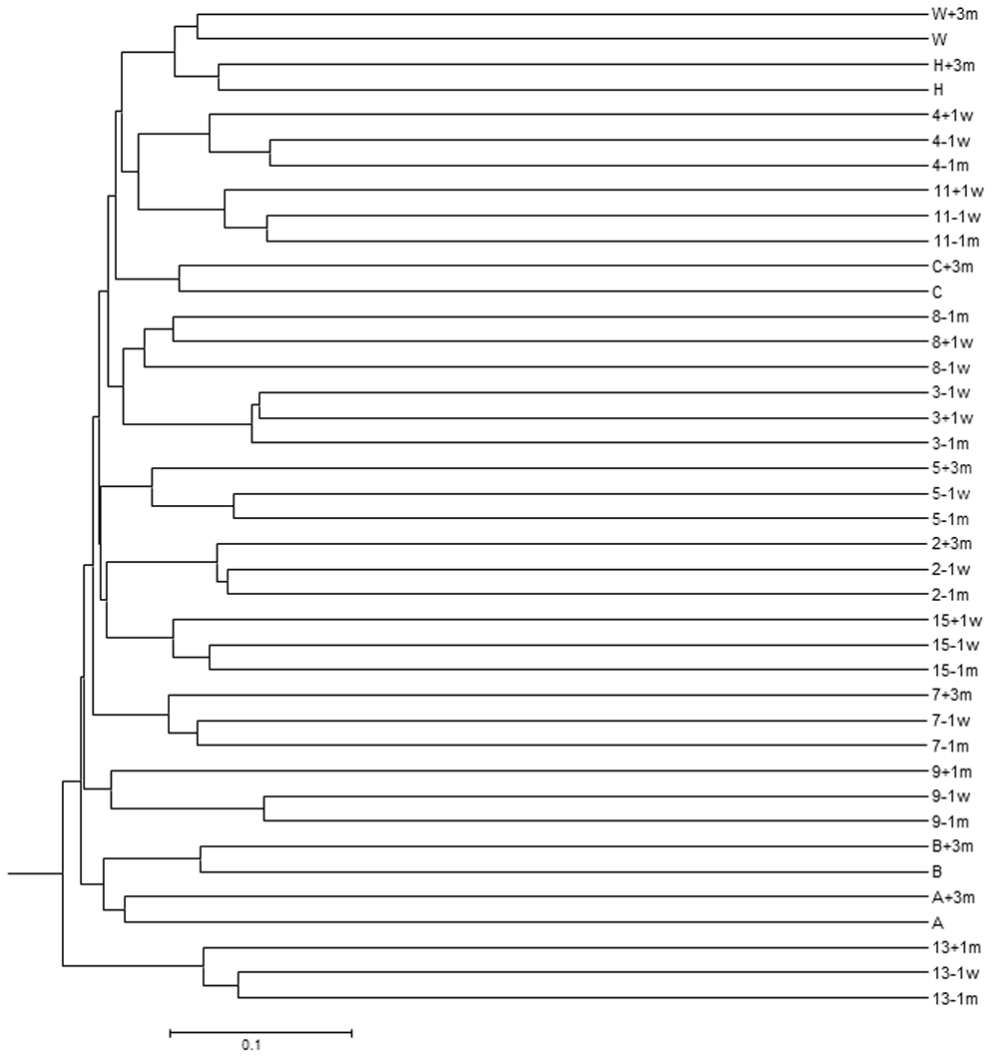
Comparison of the composition of the fecal microbial communities of each sample using the Jaccard similarity coefficient. Distances between communities, based on the presence and absence of 16S rRNA sequences, were calculated with the Jaccard coefficient (jclass) within Mothur and clustered using the UPGMA algorithim. The first numeral/letter (subjects that underwent colonoscopy/subjects who did not) corresponds to the subject, the last numeral indicates when the sample was obtained. For subjects who underwent colonoscopy: −1m, −1w, sample obtained one month, one week pre-colonoscopy, +1w, +1m, +3m, sample obtained 1 week, one month, or 3–6 months post-colonoscopy, respectively. For subjects who did not undergo colonoscopy: +3m, sample obtained 3–6 months after the first sample.

**Figure 2:**
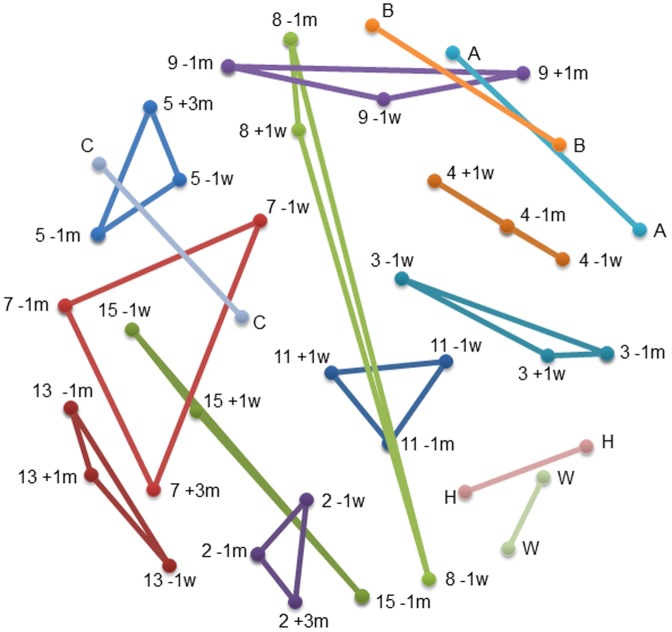
NMDS plot of the microbial communities of subjects’ samples using a distance matrix calculated with the Jaccard similarity coefficient. The plot of the two NMDS axes was generated using a distance matrix calculated at the 3% level, with the Jaccard similarity coefficient within Mothur for all samples. The distance between two points is directly proportional to the Jaccard similarity value for two samples such that sites positioned close together share more OTUs than samples further apart. NMDS stress = 0.15. Refer to Figure 1 legend for sample labelling.

**Figure 3:**
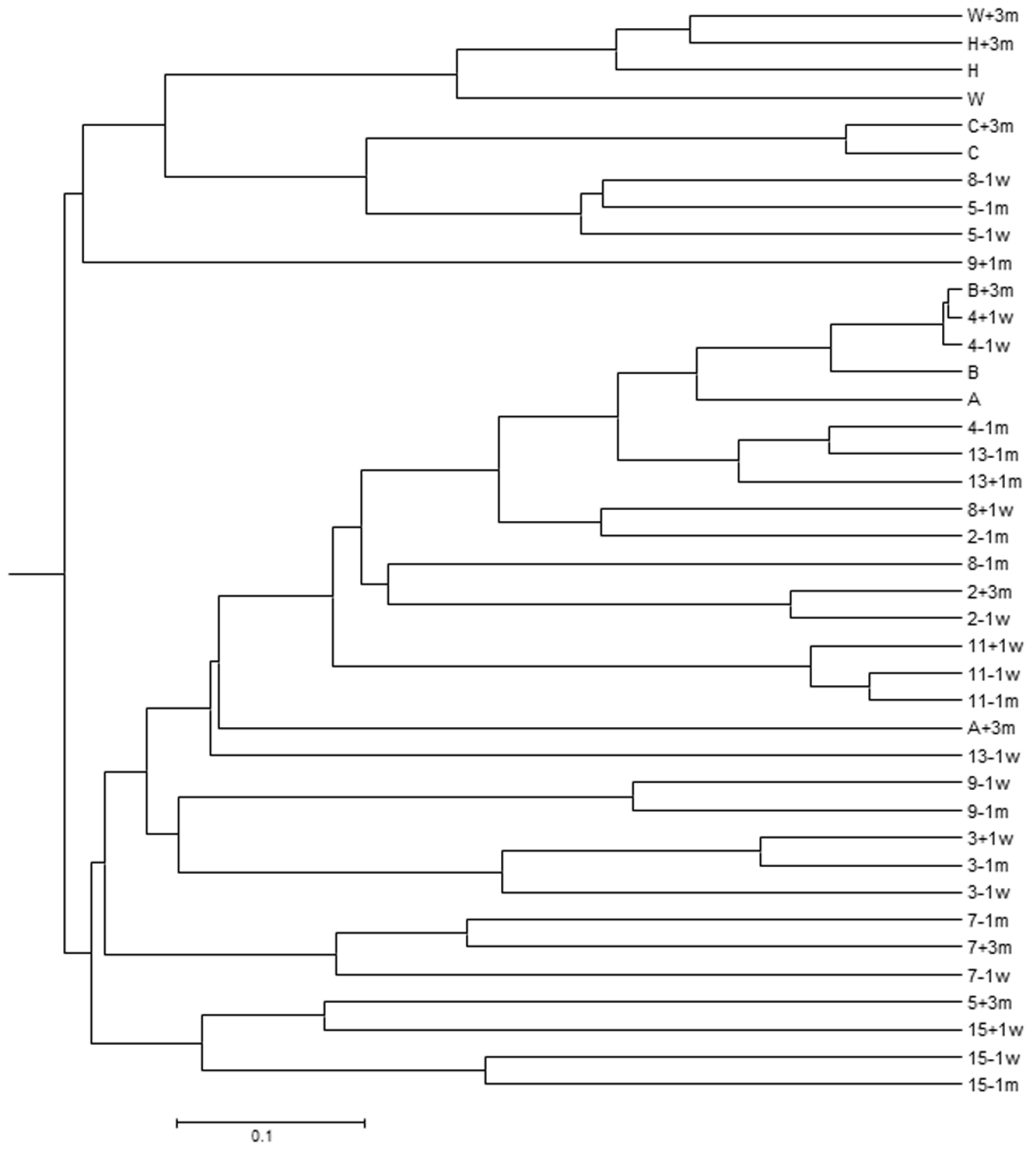
Comparison of the relative abundance of fecal microbial communities of each sample using the Yue and Clayton theta measure of dissimilarity. Dissimilarity between the relative abundance of communities was calculated with the Yue and Clayton measure of dissimilarity within Mothur and clustering performed using the UPGMA algorithm. The first numeral/letter indicates the patient or control, the last numeral indicates when the sample was obtained. Refer to Figure 1 legend for sample labelling.

**Figure 4:**
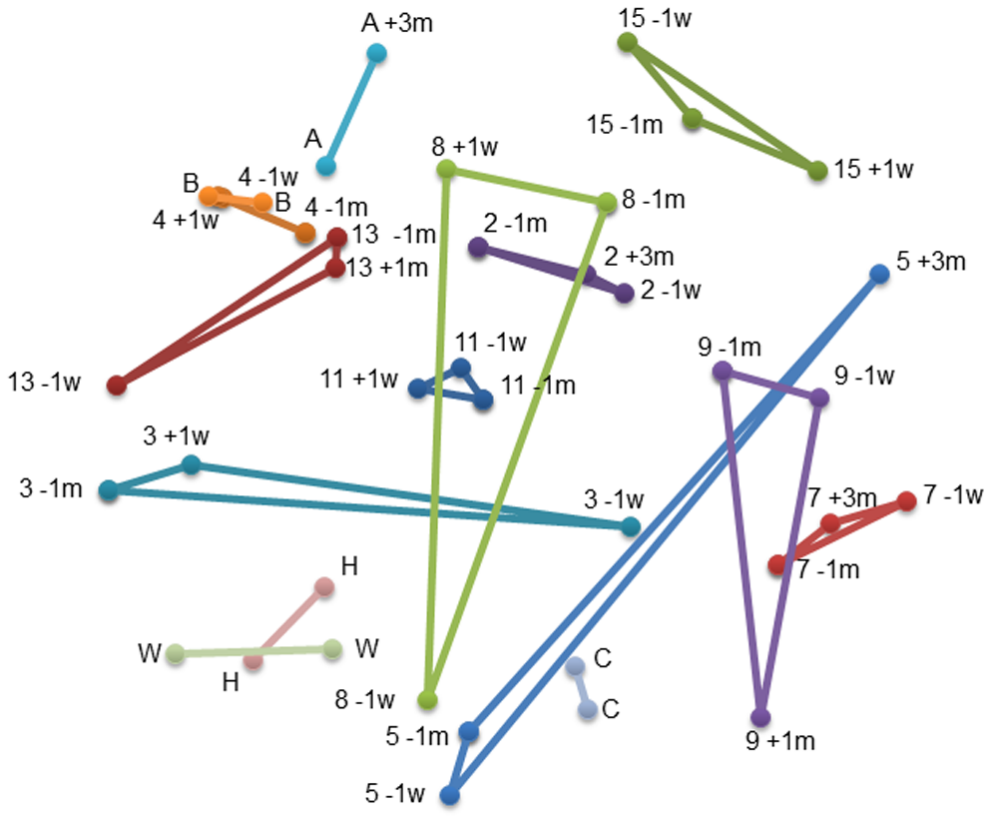
NMDS plot of the microbial 16S rRNA communities of subjects’ samples using a distance matrix calculated with the Yue and Clayton theta similarity coefficient. The plot of the two NMDS axes was generated using a distance matrix calculated at the 3% level, with the Yue and Clayton theta similarity coefficient within Mothur for all samples. The distance between two points is directly proportional to the Yue and Clayton theta similarity value for two samples such that sites positioned close together share a similar abundance in OTUs than samples further apart. NMDS stress = 0.52. Refer to Figure 1 legend for sample labelling.

The composition of the microbial communities in the subjects’ samples was compared based on the presence/absence (Jaccard coefficient, Figs. 1 and 2), and relative abundance (Yue and Clayton theta [θ_YC_], Figs. 3 and 4) of operational taxonomic units (OTU). The results of each comparison are viewed as a dendrogram (Figs. 1 and 3) and a corresponding NMDS plot (Figs. 2 and 4). NMDS is a nonparametric ordination-based method for reducing community data complexity and identifying meaningful relationships among communities.

When comparing the microbial composition of OTUs (Fig. 1), samples from the same individual are more similar and cluster together. In some subjects the pre-colonoscopy samples had the most similar microbial composition, whereas in others a pre- and post-colonoscopy sample was most similar (Fig. 2). Subjects 7, 8, and 15 had at least one sample that was was quite dissimilar.

There were few changes in the relative abundance of OTUs in the majority of subjects (Fig. 3): all samples from 4/10 subjects that underwent colonoscopy (3, 7, 11, and 15) and 1/5 subjects who did not (C) clustered together, while samples from the other subjects were in related clusters in the dendrogram. They all plotted closely together in the NMDS plot (Fig. 4), indicating minor changes in the relative abundance of OTUs.

**Figure 5:**
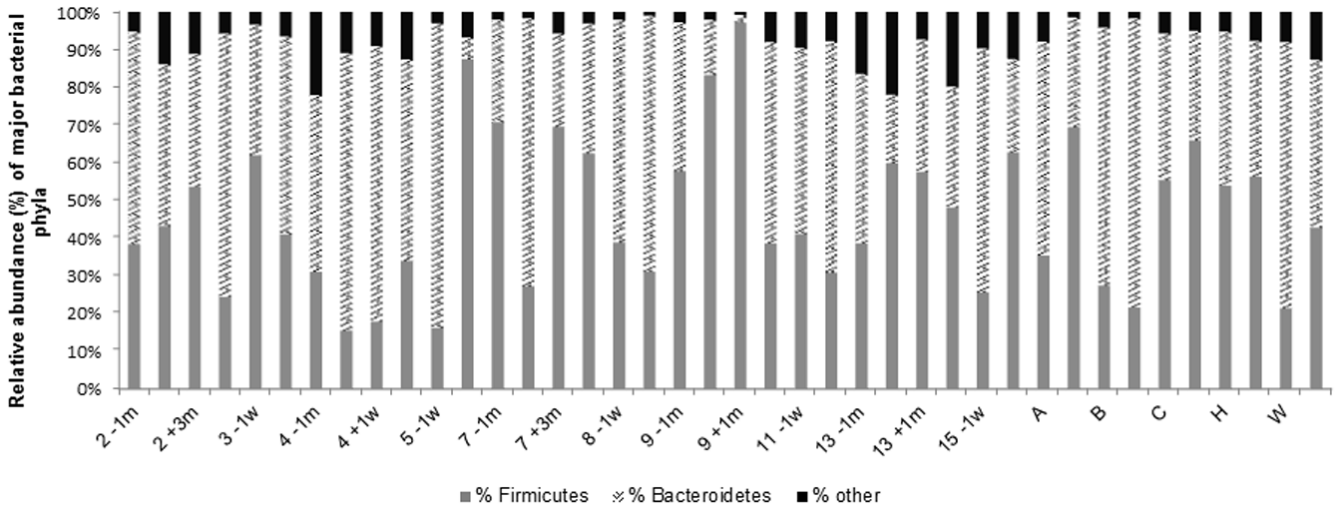
Relative abundance of *Firmicutes* and *Bacteroidetes* 16S rRNA sequences for all subjects’ samples analysed using high-throughput sequencing. Refer to Figure 1 legend for sample labelling.

In four of ten subjects (3, 5, 8, 9), one pre- or post-colonoscopy sample was quite dissimilar from the other two samples indicating differences in the relative abundance of OTUs; this was investigated by assessing the distribution of the two major phyla, *Firmicutes* and *Bacteroidetes*. The relative abundance of these two phyla varied widely among subjects (Fig. 5). A shift towards an increase in *Firmicutes* was observed in four of the five subjects who did not consume the preparation and averaged 18% (Table 3). Large shifts (>32%) in the relative proportions of *Firmicutes* and *Bacteroidetes* (Fig. 5, Table 3) were observed in comparisons involving the dissimilar samples; samples that were dissimilar to the other sample(s) from the same subject, and correlate with the results in the dendrograms and NMDS plots (Figs. 1, 2, 3, 4). To gain insight into these shifts, the raw data for the 50 predominant OTUs were compared to determine whether or not one or two OTUs could explain changes in the relative abundance in the Yue and Clayton NMDS plot. We found there were no consistent findings: different OTUs contributed to changes in the relative abundance of the dominant phyla in different subjects. For example, the changes in subject 5 are due to a decrease in OTU 2, *Bacteroides* (phyla *Bacteroidetes*), and an increase in *Rikenella* and other genera of the phyla *Firmicutes.* In subject 9 the changes are due to an increase in OTU 9, *Tannerella* (phylum *Firmicutes*), and a decrease in genera belonging to the phylum *Bacteroidetes* including *Paraprevotella.* The shift in subject A can be attributed to a decrease in OTU 1, *Bacteroides*, and an increase in OTUs belonging to the phylum, *Firmicutes.*

**Table 3:** Summary of shifts (>32%) in the relative proportions of *Firmicutes* and *Bacteroidetes* for subjects with dissimilar samples, and all shifts observed for subjects who did not undergo colonoscopy.

Subject	Indication*	Dissimilar sample**	Sample comparisons***		
			−1m *vs* −1w	−1m *vs* LS	−1w *vs* LS
3	UC	2	38 ↑	20 ↓	18 ↑
5	UC	5	22 ↓	77 ↑	55 ↑
7	AP	2	44 ↓	46 ↑	2 ↑
8	AP	2	24 ↓	9 ↓	33 ↓
9	UC	4	26 ↑	39 ↑	39 ↑
15	FHCRC, PP	2	32 ↓	44 ↑	12 ↑
A	N/A	–	32 ↑	–	–
B	N/A	–	7 ↓	–	–
C	N/A	–	10 ↑	–	–
H	N/A	–	4 ↑	–	–
W	N/A	–	26 ↑	–	–

* Abbreviations for indications for colonoscopy: UC, ulcerative colitis, AP, abdominal pain, FHCRC, family history of colorectal cancer, PP, previous polyps, N/A, did not undergo colonoscopy; indication not applicable.

** Sample most dissimilar to other sample(s) from the same subject.

*** Values showing shifts (%) in *Firmicutes* and *Bacteroidetes* phyla for samples obtained from the same patient at different time points, ↑, increase in *Firmicutes* relative to the first sample, ↓, decrease in *Firmicutes* relative to the first sample for each comparison. For subjects who underwent colonoscopy: −1m, −1w, sample obtained one month, one week pre-colonoscopy, and LS refers to the last sample obtained post-colonoscopy (refer to Table 3), respectively. For subjects who did not undergo colonoscopy: +3m, sample obtained 3–6 months after the first sample.

**Figure 6:**
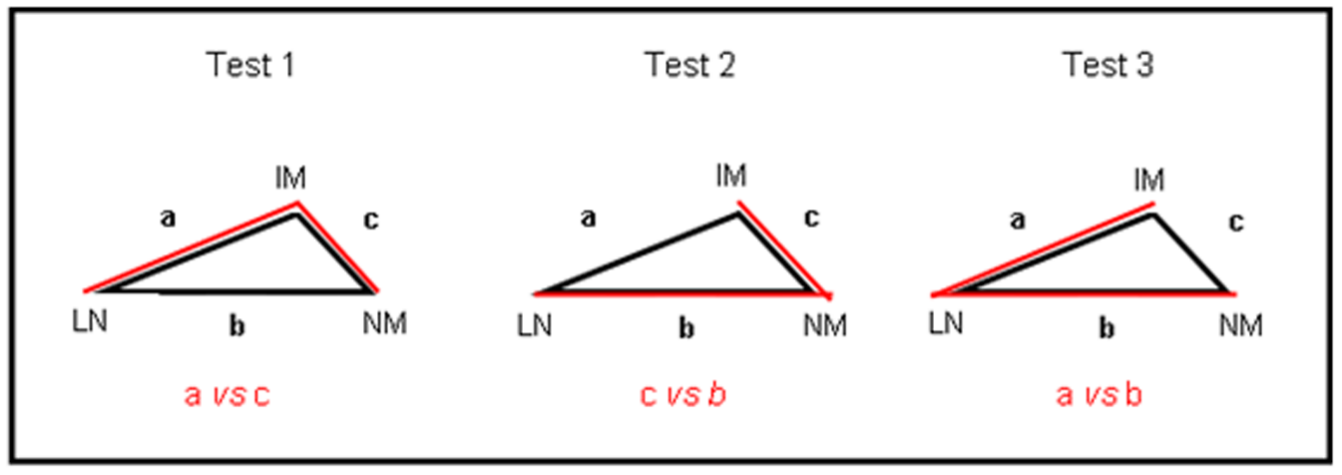
Three tests used for the matched-pairs *t*-test analyses for three different combinations of the three samples analysed using HTS for subjects who underwent colonoscopy. −1m, −1w, sample obtained 1 month, 1 week pre-colonoscopy, LS, the last post-colonoscopy sample obtained for each patient. Test 1 compares the mean distances of a and c, test 2, c and b, and test 3, a and b.

Because samples from the same subject were expected to be more similar than samples from another subject, standard analyses could not be employed; the distances between samples in the two NMDS plots were compared using matched pairs *t*-tests (Fig. 6 and Table 4). There were no significant differences in the distance between samples obtained in the absence of colonoscopy preparation and the distance between samples obtained pre- and post-colonoscopy. We conclude that colonoscopy preparation with PEG does not affect the microbiota of subjects to a greater degree than the temporal variation seen in those who did not undergo the preparation.

**Table 4:** Comparison of the mean distances between samples observed in the presence/absence (Jaccard) or relative abundance (Yue and Clayton) NMDS plots using matched pairs *t*-tests.

Basis of comparison	P value of sample comparison		
	−1m *vs* −1w	−1m *vs* LS	−1w *vs* LS
Jaccard	0.65	0.12	0.85
Yue and Clayton	0.11	0.22	0.06

For subjects who underwent colonoscopy: −1m, −1w, sample obtained one month, one week pre-colonoscopy, +1w, +1m, +3m, sample obtained 1 week, one month, or 3–6 months post-colonoscopy, respectively. For subjects who did not undergo colonoscopy: +3m, sample obtained 3–6 months after the first sample. Figure 5 outlines the sample comparisons for each test. P = <0.05.

## Discussion

The composition of the microbiota was compared in fecal samples collected pre- and post-colonoscopy in subjects using PCR-DGGE (14 subjects) and HTS (10 subjects). Two fecal samples obtained 3–6 months apart were collected from five healthy subjects who did not undergo the procedure and were analysed by both methods.

There was greater than 85% similarity among the DGGE profiles of samples obtained in the absence of colonoscopy preparation suggesting that temporal variation occurred naturally. For the majority of subjects (11/14) who consumed the preparation, no significant changes were observed in the pre- and final post-colonoscopy PCR-DGGE comparisons. The three samples with less than 85% similarity may still reflect temporal changes. Caporaso *et al.* [2] took daily stool samples from two healthy subjects for 6 and 15 months and showed that the gut microbiota has a relatively small temporal core; approximately 5% of OTUs at the 97% OTU level were present in 95% of samples.

Analysis of our HTS results indicated that the fecal microbiota of all subjects, varied in presence/absence and/or abundance of different OTUs whether they consumed the preparation or not. The greatest variation could be explained by differences in abundance of genera belonging to the main gut phyla, *Bacteroidetes* and *Firmicutes*. Variations were not statistically significant when compared to those observed in the absence of colonoscopy preparation. Because we did not include a bead-beating step in the extraction protocol, we may have failed to lyse and therefore detect a minor subset of microbes, however our protocol was consistent across subject and control samples. Collectively, our results suggest that colonoscopy does not affect the gut microbiota.

Only two other studies investigated the effect of colonoscopy on the gut microbiota [12], [13]. Mai *et al*. [12] used PCR-DGGE and 16S rRNA gene *E. coli*-clone libraries to compare a pre-colonoscopy sample with two post-colonoscopy samples obtained 2–4 and 6–8 weeks post-colonoscopy; no controls were included. The DGGE results indicated that for 3/5 patients the post-colonoscopy samples were more similar to each other than the pre-colonoscopy sample. For the other two patients, little difference was seen between the pre- and post-colonoscopy samples. Analysis of the *E. coli* clone libraries suggested that all three samples from each patient were related to varying degrees but in many cases the degree of relatedness was different to that observed in DGGE. Despite the incongruence of results using different methods and not having factored in normal temporal variation, the authors concluded that the microbiota composition is disturbed in patients undergoing screening colonoscopy in this small study.

Harrell *et al.* [13] also used 16S rRNA gene *E. coli* clone libraries to investigate short-term effects on microbiota diversity, richness, and composition of biopsies obtained from healthy individuals.In the first phase of the study, five subjects underwent two sigmoidoscopies over two weeks. The first was performed without any preparation, the second preceded by a clear liquid diet for 24 hours and a standard (PEG) bowel preparation. The results were divergent: diversity increased in three subjects and decreased in two. In the second phase of the study, all subjects underwent the first procedure without any preparation, but the second procedure was performed less than one week later and was preceded by no preparation for two subjects (control group 1), a 24 h liquid diet for another two subjects (control group 2), and a 24 h liquid diet and PEG preparation for the remaining three patients (experimental group). An average of 225 16S rRNA gene sequences were obtained for each biopsy. The diversity and richness of the microbiota of the second biopsy from the experimental group was lower than that of the first biopsy, and the composition of the microbiota in the two samples differed at the genus level. There were no significant differences in any of these parameters between the two biopsies in the control groups. The authors concluded that the PEG preparation, not the procedure or liquid diet, may significantly alter the colonic microbiota in the short-term.

Like Mai *et al.* [12], we also saw discrepant results using different techniques: the NMDS plot indicated that dissimilar samples identified by PCR-DGGE were not necessarily dissimilar using HTS analysis (Figs. 1, 2, 3, 4). Conversely, results showing similarity using DGGE were not necessarily so using HTS (e.g. samples −1m and −1w, Table 1). Some samples were dissimilar by both methods (e.g. samples −1m and +3m of subject 9 and samples −1w and +3m of subject 5, Table 1). There are a number of possible reasons for the discrepancies in the DGGE and HTS analyses. DGGE detects only the most abundant species: an average of 26 bands was observed for each DGGE profile, in contrast to an average of 504 OTUs per sample using HTS. While in theory each band in DGGE represents a single species, different species may migrate to the same position and the same species may migrate to different positions (for examples, see [24], [25]). Unless cloning and sequencing is used, identification of PCR artifacts such as chimeras is not possible using DGGE, and sequence libraries may contain a few to more than 45% chimeric sequences [26], [27]. Nevertheless, DGGE is inexpensive and when used carefully, produces meaningful results.

We sought to determine whether or not there were clinical factors that may explain the findings. For example, subjects 3, 5 and 9, all had ulcerative colitis that was quiescent at the time of the study, yet were identified by one or both methods as having a dissimilar sample (Tables 1 and 3, and data not shown). For subjects 5 and 9, the last post-colonoscopy sample was dissimilar suggesting that the preparation may have had a lasting effect on the microbiota. In patients suffering from ulcerative colitis, colonoscopy results in worsening symptoms and an increased need for medications in one in eight and one in ten patients respectively [28]. Whether this is due to a direct effect of the preparation on the mucosa, or an indirect effect on the gut microbiota, or other factors, is not known. Investigators using biopsy samples to examine mucosa-associated bacteria report an increase in *Bacteroidetes* and decrease in *Firmicutes* [29], [30] in ulcerative colitis patients, but our study shows an increase in the proportion of *Firmicutes* (Fig. 5, Table 3) consistent with the findings of Takaishi et al [31] who also examined stool samples. Mucosa-associated bacteria are known to differ from fecal bacteria [1], [32], so it is not possible to compare the findings of studies using different sites for sampling. Medications may also result in changes in the microbiota: subject 4 had been on iron supplements until 10 days prior to the procedure, which may account for the changes seen post-procedure.

Our study highlights the difficulties in studying the intestinal microbiota: we saw temporal changes in all subjects and disparate results from two commonly used analytical tools. We interpret our results as showing that any differences in the fecal microbiota before and after colonoscopy are no greater than those seen in normal subjects over time. We conclude that consumption of PEG does not have a significant impact on gut microbiota in the majority of subjects, although a minority of subjects with a microbiota that exhibits greater natural temporal variation may be more susceptible to perturbations. It remains to be determined whether or not patients suffering from diseases associated with possible dysbiosis of the gut microbiota, such as ulcerative colitis, are more susceptible to persisting perturbations from physical effects such as colonoscopy preparation.

## Supporting Information

Dataset S1 All sff files generated by the 454 Genome Sequencer, their associated metadata, and primer sequences are available via the following link: https://datacommons.anu.edu.au:8443/DataCommons/item/anudc:4896.
(XLSX)
